# Spectrum and antibiotic sensitivity of bacterial keratitis: a retrospective analysis of eight years in a Tertiary Referral Hospital in Southwest China

**DOI:** 10.3389/fcimb.2024.1363437

**Published:** 2024-03-11

**Authors:** Rui-Qin Guo, Ji Yang, Ya-Bin Yang, Ya-Nan Chen, Yu-Yuan Xiao, Ping Xiang, Meng-Jie Dong, Min-Fang He, Yin-Ting Wang, Yun-Ling Xiao, Hong-Qin Ke, Hai Liu

**Affiliations:** ^1^ Department of Ophthalmology, Second People’s Hospital of Yunnan Province, The Affiliated Hospital of Yunnan University, The Eye Disease Clinical Medical Research Center of Yunnan Province, The Eye Disease Clinical Medical Center of Yunnan Province, Kunming, China; ^2^ Yunnan Province Innovative Research Team of Environmental Pollution, Food Safety, and Human Health, Institute of Environmental Remediation and Human Health, School of Ecology and Environment, Southwest Forestry University, Kunming, China; ^3^ Department of Ophthalmology, Honghe County People’s Hospital, Honghe, China

**Keywords:** bacterial keratitis, retrospective analysis, pathogenesis, drug susceptibility, risk factors

## Abstract

**Purpose:**

The objective of this study was to investigate the epidemiological characteristics, distribution of isolates, prevailing patterns, and antibiotic susceptibility of bacterial keratitis (BK) in a Tertiary Referral Hospital located in Southwest China.

**Methods:**

A retrospective analysis was conducted on 660 cases of bacterial keratitis occurring between January 2015 and December 2022. The demographic data, predisposing factors, microbial findings, and antibiotic sensitivity profiles were examined.

**Results:**

Corneal trauma emerged as the most prevalent predisposing factor, accounting for 37.1% of cases. Among these cases, bacterial culture results were positive in 318 cases, 68 species of bacteria were identified. The most common Gram-Positive bacteria isolated overall was the *staphylococcus epidermis* and the most common Gram-Negative bacteria isolated was *Pseudomonas aeruginosa*. Methicillin-Resistant *Staphylococci* accounted for 18.1% of all Gram-Positive bacteria. The detection rate of *P. aeruginosa* showed an increasing trend over time (*Rs=0.738, P=0.037*). There was a significant decrease in the percentage of Gram-Negative microorganisms over time (*Rs=0.743, P=0.035*). The sensitivity of Gram-Positive bacteria to linezolid, vancomycin, tigecycline, quinupristin/dalfopristin, and rifampicin was over 98%. The sensitivity rates of Gram-Negative bacteria to amikacin, meropenem, piperacillin/tazobactam, cefoperazone sodium/sulbactam, ceftazidime, and cefepime were all above 85%. In patients with a history of vegetative trauma, the possibility of BK should be taken into account in addition to the focus on fungal keratitis.

**Conclusion:**

The microbial composition primarily consists of Gram-Positive cocci and Gram-Negative bacilli. Among the Gram-Positive bacteria, *S. epidermidis* and *Streptococcus pneumoniae* are the most frequently encountered, while *P. aeruginosa* is the predominant Gram-Negative bacteria. To combat Gram-Positive bacteria, vancomycin, linezolid, and rifampicin are considered excellent antimicrobial agents. When targeting Gram-Negative pathogens, third-generation cephalosporins exhibit superior sensitivity compared to first and second-generation counterparts. As an initial empirical treatment for severe cases of bacterial keratitis and those unresponsive to fourth-generation fluoroquinolones in community settings, the combination therapy of vancomycin and tobramycin is a justifiable approach. Bacterial keratitis can be better managed by understanding the local etiology and antibacterial drug susceptibility patterns.

## Introduction

Bacteria have a wide distribution in soil and water, and they also exist symbiotically with other organisms. The human body carries a substantial amount of bacteria. It is estimated that the total number of bacterial cells in the human body, including the epidermis, is approximately ten times the total number of human cells. Bacteria are ubiquitous, and the healthy ocular surface microbiome can be classified into 12 phyla, 70 genera, and 140 species. Among the species with high relative abundances and high positivity rates on the ocular surface are *Streptococcus pyogenes*, *S. epidermidis*, *Propionibacterium acnes*, and so on (Kang et al., 2021). A healthy ocular surface can live in harmony with symbiotic microorganisms. However, when the corneal epithelial barrier is compromised by trauma, diseases, or medications, external bacteria may invade the cornea and lead to bacterial keratitis (BK).

Bacterial keratitis is a prevalent cause of vision loss (Ung and Chodosh, 2021), causing blindness in up to 2 million eyes worldwide each year (Ung et al., 2019). It is an ophthalmic emergency that necessitates immediate symptomatic management. Bacterial keratitis can manifest as a slowly progressing ulcer or as a septic infection with rapid deterioration of corneal tissue. Poorly controlled infection can result in progressive tissue destruction and severe visual impairment (Durand et al., 2021). Common microorganisms responsible for bacterial keratitis include *S. aureus*, coagulase-negative *staphylococci* (CoNS), and *S. pneumoniae* (Prokosch et al., 2012; Ahmed et al., 2022; Zhang et al., 2022). Corneal ulcers caused by bacterial infestation are often associated with persistent chronic ocular surface diseases, long-term corneal contact lens wear, eye trauma, and systemic or local corneal immunosuppression (Amarasekera et al., 2019; Durrani et al., 2019, 2020; Verner et al., 2020). The spectrum of pathogens can vary depending on the geographical location, local environment, and climatic conditions (Satpathy et al., 2019).

There is a paucity of information regarding a continuous retrospective analysis of the pathogenic spectrum and drug susceptibility of bacterial keratitis in southwest China. Understanding the recent local epidemiological patterns of pathogens and their susceptibility profiles may provide evidence-based guidelines for the successful treatment of bacterial keratitis (Estopinal and Ewald, 2016; Acharya et al., 2020). Thus, this study aims to review the epidemiological characteristics of bacterial keratitis, trends in corneal isolates, and their susceptibility to commonly used antimicrobial drugs over an eight-year period in a tertiary referral hospital in the southwest region.

## Materials and methods

### Patients

This was a retrospective review of medical case records and microbiological records of all patients from January 1, 2015, through December 31, 2022, at Eye Hospital, Yunnan, China. It was commenced after receiving clearance from the Institute Ethics Committee of the Affiliated Hospital of Yunnan University. Due to the retrospective nature of the study, ethics approval was given with a waiver for “Informed Consent of the patient”.

We evaluated 2564 patients who had clinical suspicion of corneal ulcerations. A corneal ulcer was defined as a loss of corneal epithelium with a stromal infiltrate and suppuration that are associated with signs of inflammation, with or without hypopyon. A standardized form was filled out for each patient, documenting the patient’s sociodemographic information, the duration of symptoms, predisposing factors, any history of corneal trauma and trauma-causing agents, any associated ocular conditions, other systemic diseases, treatments received before presentation and microbial results.

### Clinical examinations

All patients received a slit lamp biomicroscopic and AC-OCT examination by experienced ophthalmologists. Clinical features such as the size and depth of the stromal infiltrate, the size of the ulcer (measured in millimeter), the presence or absence of a hypopyon (measured in millimeters), pre-existing viral keratitis, and chronic corneal disease were noted. Also, we noted the use of contact lenses, history of corneal trauma, previous ocular surgery, as well as other systemic combinations.

### Laboratory investigation

Corneal scrapings were obtained using a sterile blade (No. 15), and specimens were sent immediately after collection for corneal ulcer culture plus drug sensitivity and fungal smear examination. A part of the sample was smeared onto a glass slide for Gram staining to check bacteria and Melan stain for detecting fungi. The rest of the samples were immediately inoculated in pediatric bottles on a Bact/Alert3D blood culture instrument, placed in a fully automated incubator, and positive cultures were transferred to, MacConkey agar and Sabro agar for isolation and culture using VITEK-2Compact for strain identification, the cultured microorganisms were identified using standard microbiological procedures.

Drug susceptibility testing was performed according to the Clinical Laboratory Standards Association’s antibiotic susceptibility testing standards. Antimicrobial susceptibility testing for bacterial isolates was performed using the Kirby–Bauer disk diffusion method. The antibiotic discs were used as per the recommendations of the Clinical and Laboratory Standards Institute (CLSI) for a particular group of bacteria.

### Statistical analyses

Demographic and microbiological details of the cases were entered in Microsoft Excel sheets. Statistical analyses were performed using SPSS software version 22 (SPSS Inc, Chicago, IL). Descriptive statistics and means were used for continuous variables; ratios and percentages of categorical variables were used to describe the sample. Cardinality tests were used for data analysis, and Spearman’s rank correlation coefficient was used to test for trends. *P < 0.05* was considered significant.

## Results

### Demographics and predisposing factors

Amongst the cohort of 2,564 keratitis patients admitted to tertiary eye centers between January 2015 and December 2022, a noteworthy subset of 660 individuals (25.7%) were diagnosed with bacterial keratitis (BK). Within this subset, 434 patients (65.8%) presented solely with bacterial infection, while 226 patients (34.2%) exhibited a confluence of bacterial and other types of keratitis. The average age of the participants in this study was 55 years (range: 45-65), with the most prevalent age group falling between 40 and 65 years (373 patients, 56.5%). There were 396 male patients (60.0%) and 264 female patients (40.0%). Notably, farmers constituted the majority of cases at 425 (64.4%), while workers accounted for 16 cases (2.4%), and individuals in various other occupations represented 219 cases (33.2%).

In our study, corneal trauma emerged as the most prevalent risk factor, accounting for 245 cases (37.1%). Among these cases, 70 individuals had a history of recurrent keratitis, 39 had ocular surface disease, and 14 had undergone ophthalmic surgeries such as pterygium surgery, penetrating corneal transplantation, or cataract surgery. Interestingly, only 4 cases had a history of contact lens wear, while the remaining cases did not exhibit any localized risk factors. Additionally, 66 cases reported the presence of systemic risk factors. Specifically, diabetes mellitus was identified in 37 cases, autoimmune diseases in 21 cases, cold-induced keratitis in 6 cases, and human immunodeficiency virus (HIV) positivity in 2 cases.

### Clinical characteristic

Corneal ulcers were observed to have a diameter of ≤2 mm in 32 cases (4.8%), ranging from 2 to 5 mm in diameter in 355 cases (53.8%), and exceeding 5 mm in diameter in 273 cases (41.4%). In terms of depth, 382 cases (57.9%) were classified as deep ulcers, 262 cases (39.7%) as medium-depth ulcers, and 16 cases (2.4%) as superficial ulcers. The severity of bacterial keratitis (BK) patients was graded based on the extent and depth of corneal ulceration. Mild corneal ulceration was identified in only 0.5% (3/660) of patients, moderate corneal ulceration in 32.3% (213/660) of patients, and severe corneal ulceration in the majority, accounting for 67.3% (444/660) of patients. Notably, 48.5% (320/660) of patients exhibited hypopyon, and ulcer perforation was observed in 28.0% (185/660) of patients.

For a comprehensive presentation of the demographic features, risk factors, clinical manifestations, and progression of these keratitis cases, please refer to **Table 1**.

**Table 1:** Demographics, Risk Factors, and Clinical Characteristic Features of Patients.

	Details	Total Patients(n)	Frequency (%)
Age (in years)	<12	39	5.9
	12-18	5	0.8
	18-40	68	10.3
	40-65	373	56.5
	>65	175	26.5
Sex			
	Male	396	60.0
	Female	264	40.0
Symptom duration(days)			
	1-7	126	19.1
	7-30	251	38.0
	>30	283	42.9
Occupation			
	Farmer	425	64.4
	Worker	16	2.4
	Other Occupations	219	33.2
Size of ulcer			
	≤2mm	32	4.8
	2-5mm	355	53.8
	>5 mm	273	41.4
Corneal ulcer depth			
	deep	382	57.9
	medium depth	262	39.7
	superficial	16	2.4
Severity			
	mild	3	0.5
	moderate	213	32.3
	severe	444	67.2
hypopyon			
	Yes	320	48.5
	No	340	51.5
Ulcer perforation			
	Yes	185	28.0
	No	475	72.0
Risk factors			
	Corneal trauma	245	37.2
	Contact lens	4	0.6
	Preexisting keratitis	70	10.6
	Previous ocular surgery	14	2.1
	Ocular Surface Diseases	39	5.9
	No obvious cause	288	43.6

### Microbiological culture findings

A microbiological examination was conducted on 632 eye specimens, and 318 (50.3%) of the smears tested positive, resulting in a total of 330 positive strains being detected. Among the 318 patients with positive cultures, 5 cases (1.6%) exhibited co-infection with both Gram-Positive and Gram-Negative bacteria, while 7 cases (2.2%) showed co-infection with only Gram-Positive bacteria. The cultured bacteria encompassed a diverse range of 68 strains, consisting of 260 (78.8%) Gram-Positive strains and 70 (21.2%) Gram-Negative strains. Among these, the predominant strains were 231 (70.0%) Gram-Positive cocci and 69 (20.9%) Gram-Negative bacilli. The Gram-Positive cocci were predominantly identified as *Staphylococcus* spp., accounting for 139 strains (60.2%), followed by *Streptococcus* spp. with 70 strains (30.3%). Additionally, *Micrococcus* spp. was observed in 17 strains (*Micrococcus luteus*), and *Enterococcus* spp. was detected in 5 strains. As for the Gram-Negative bacilli, *Pseudomonas* spp. accounted for the majority with 24 strains (34.8%), followed by *Klebsiella* spp. with 7 strains (10.1%). Other strains included *Escherichia* spp. with 6 strains (*Escherichia coli*), *Serratia* spp. with 5 strains (*Serratia marcescens*), *Bacillus* spp. and *Enterobacter* spp. with 3 strains each, *Bacillus* spp. with 2 strains, and single strains of *Aspergillus* spp. and *Moraxella* spp. (**Table 2**).

**Table 2:** Microbiological Culture Findings.

Bacteria	N	%
Gram-Positive cocci *Staphylococcus* spp.	231 139	70.0 42.1
*Streptococcus* spp.	70	21.2
*Micrococcus* spp.	17	5.2
*Enterococcus*	5	1.5
Gram-Negative bacillus	69	20.9
*Pseudomonas* spp.	24	7.3
*Klebsiella* spp.	7	2.1
*Ehr*lichia spp.	6	1.8
*Serratia* spp.	5	1.5
*Bacillus* spp.	3	0.9
*Enterobacter* spp.	3	0.9
*Alkali-producing Bacillus* spp.	2	0.6
*Bacillus variegatus* spp.	1	0.3
*Moraxella* spp.	1	0.3
Other	17	5.2
Gram-Positive bacillus	29	8.8
*Bacillus* spp.	7	2.1
*Corynebacterium* spp.	7	2.1
Other	15	4.6
Gram-Negative cocci	1	0.3
Total bacterial isolates	330	100.0

**Figure 1:**
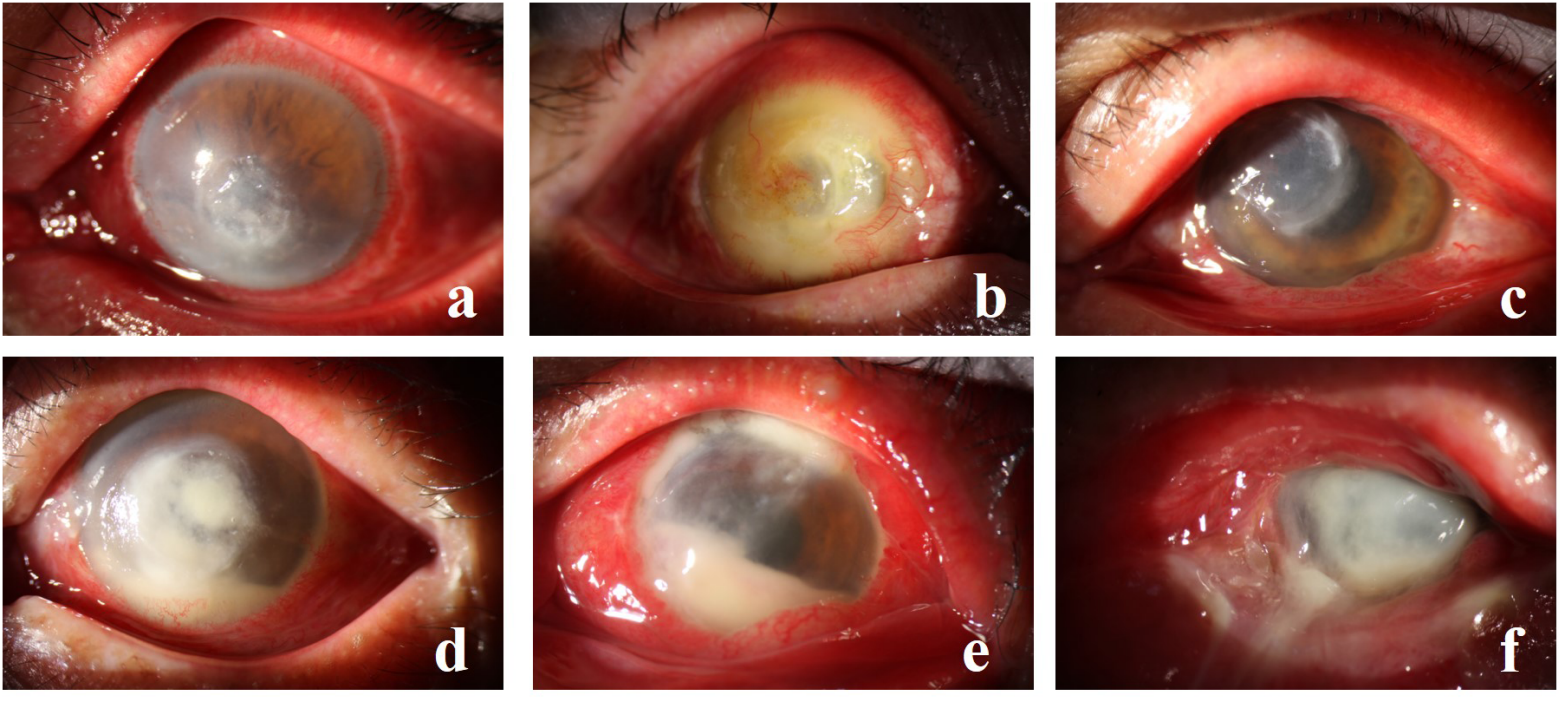
**(A, B)** depict *S. epidermidis* infection, **(C)** illustrates *S. pneumoniae*, **(D)** showcases *human Staphylococcus* with fungal infection, **(E)** displays *P. aeruginosa* infection, and **(F)** exhibits *P. aeruginosa* with *S. aureus* infection.

Among the detected strains, the top 6 most prevalent species were as follows: *S. epidermidis* (**Figures 1A, B**) at 20.9% (69/330), *S. pneumoniae* (**Figure 1C**) at 12.1% (40/330), *P. aeruginosa* (**Figures 1E, F**) at 7.0% (23/330), *human Staphylococcus* (**Figure 1D**) and *Micrococcus luteus* at 5.2% (17/330), and *streptococcus mitis* at 3.6% (12/330).

**Figure 2:**
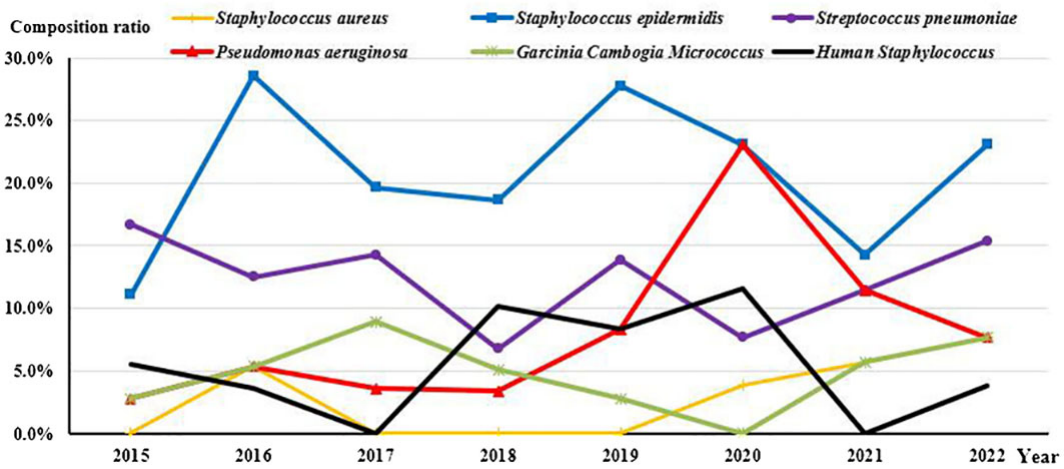
Annual changes in the composition ratio of common strains.

Since 2016, *S. epidermidis* has consistently exhibited the highest detection rate. Notably, the detection rate of *P. aeruginosa* demonstrated an increasing trend over time (*Rs=0.738, P=0.037*), while there were no significant changes observed among the other common species (**Figure 2**).

**Figure 3:**
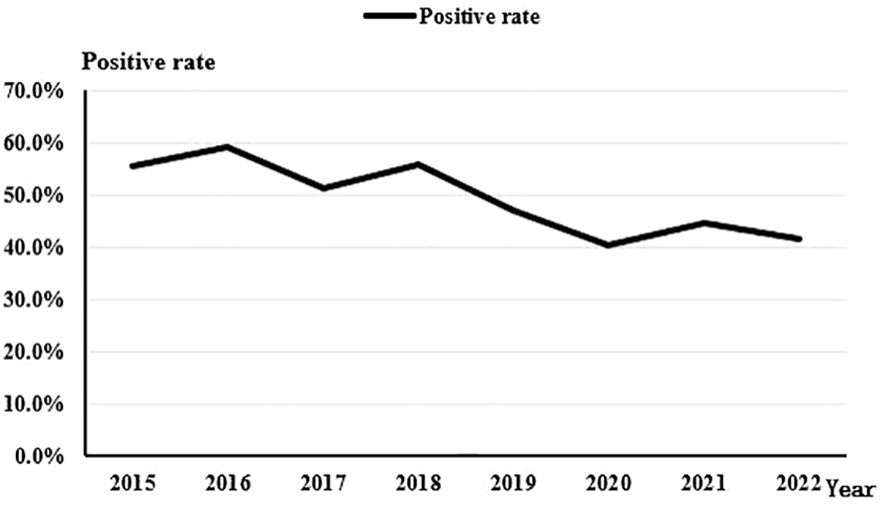
The trend of positive bacterial culture rate from 2015 to 2022.

Throughout the 8-year study period, there was a discernible downward trend in the overall positivity rate of bacterial culture from corneal scrapings (*Rs=-0.810, P=0.015*). The rate decreased from 55.4% in 2015 to 41.7% in 2022 (**Figure 3**).

**Figure 4:**
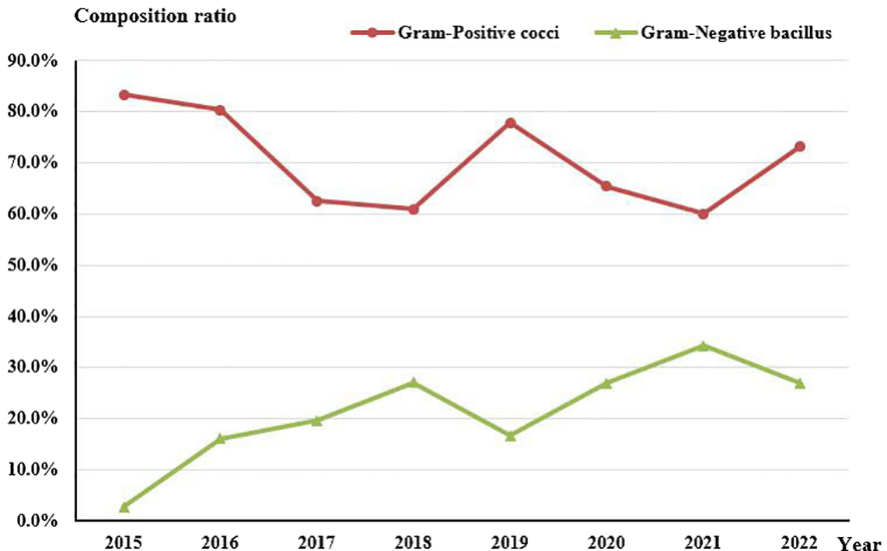
Composition of Gram-Positive cocci and Gram-Negative bacilli in culture-positive specimens from 2015 to 2022.

Concerning the composition of bacterial species, no statistically significant change was observed in the proportion of Gram-Positive cocci (*Rs=-0.524, P=0.183*). However, there was an evident increasing trend in the proportion of Gram-Negative rods (*Rs=0.743, P=0.035*) over the course of the study period (**Figure 4**).

### Antibiotic susceptibility of the bacterial strains isolated from corneal lesions

Sensitivity to antibiotics of Gram-Positive Microorganisms in **Table 3**. Sensitivity to antibiotics of Gram-Negative Microorganisms in **Table 4**. The drug susceptibility rates of common Gram-Positive bacteria can be found in **Table 5**, while the drug susceptibility rates of common Gram-Negative bacteria are also provided in **Table 6**.

**Table 3:** Sensitivity to Antibiotics of Gram-Positive Microorganisms [%/(N)].

Antibiotic	Sensitive	Resistant	Intermediary
Penicillin	44.9 (83)	54.0 (100)	1.1 (2)
Benzoxicillin	50.8 (60)	49.2 (58)	0.0 (0)
Linezolid	99.4 (159)	0.6 (1)	0.0 (0)
Vancomycin	99.5 (184)	0.5 (1)	0.0 (0)
Tigecycline	100.0 (121)	0.0 (0)	0.0 (0)
Gentamicin	75.2 (91)	5.0 (6)	19.8 (24)
Quinupristin/Dafopristin	97.7 (170)	2.3 (4)	0.0 (0)
Ciprofloxacin	65.0 (78)	27.5 (33)	7.5 (9)
Levofloxacin	74.1 (137)	24.8 (46)	1.1 (2)
Moxifloxacin	71.2 (99)	7.9 (11)	20.9 (29)
Erythromycin	24.3(45)	74.6 (138)	1.1 (2)
Clindamycin	38.0 (68)	61.4 (110)	0.6 (1)
Rifampin	98.5 (128)	0.75 (1)	0.75 (1)
Cotrimoxazole	54.5 (85)	44.2 (69)	1.3 (2)
Cefotaxime	98.4 (61)	1.6 (1)	0.0 (0)
Chloromycin	90.3 (56)	9.7 (6)	0.0 (0)

**Table 4:** Sensitivity to Antibiotics of Gram-Negative Microorganisms[%/(N)].

Antibiotic	Sensitive	Resistant	Intermediary
Cefoperazone sodium sulbactam	88.9 (48)	5.55 (3)	5.55 (3)
Piperacillin/tazobactam	90.6 (48)	7.5 (4)	1.9 (6)
Aminotransol	72.9 (35)	22.9 (11)	4.2 (2)
Ampicillin	16.7 (7)	80.9 (34)	2.4 (1)
Amoxicillin Clavulanic Acid	30.4 (14)	67.4 (31)	2.2 (1)
Piperacillin	79.55 (35)	15.9 (7)	4.55(2)
Cefazolin	16.7 (7)	80.9 (34)	2.4 (1)
Ceftazidime	85.7 (48)	14.3 (8)	0.0 (0)
Ceftriaxone	44.7 (21)	51.0 (24)	4.3 (2)
Cefepime	87.3 (48)	7.3 (4)	5.4 (3)
Ertapenem	69.7 (23)	30.3 (10)	0.0 (0)
Imipenem	83.3 (45)	9.3 (5)	7.4 (4)
Meropenem	92.7 (51)	7.3 (4)	0.0 (0)
Amikacin	87.3 (48)	12.7 (7)	0.0 (0)
Gentamicin	76.7 (33)	23.3 (10)	0.0 (0)
Ciprofloxacin	68.8 (33)	22.9 (11)	8.3 (4)
Levofloxacin	77.6 (45)	17.2 (10)	5.2 (3)
Furantoin	12.8 (5)	79.5 (31)	7.7 (3)
Cotrimoxazole	46.3 (25)	53.7 (29)	0.0 (0)
Tetracycline	25.0 (10)	75.0 (30)	0.0 (0)

**Table 5:** The antibiotic susceptibility of common Gram-Positive bacteria [%/(N)].

Antibiotic	*S. epidermidis*(n=61)	*S. aureus*(n=8)	*P. aeruginosa*(n=39)	
Penicillin	9.8 (6)	8 (100.0)	89.7 (35)
Benzoxicillin	27.9 (17)	62.5 (5)	
Linezolid	100.0 (61)	100.0 (8)	
Vancomycin	100.0 (61)	100.0 (8)	100.0 (39)
Tigecycline	100.0 (61)	100.0 (8)	
Gentamicin	65.6 (40)	50.0 (4)	
Quinupristin/Dafopristin	98.4 (60)	50.0 (4)	
Ciprofloxacin	49.2 (30)	100 (8)	
Levofloxacin	49.2 (30)	50.0 (4)	97.4 (38)
Moxifloxacin	49.2 (30)	75.0 (6)	
Erythromycin	21.3 (13)	12.5 (1)	12.8 (5)
Clindamycin	39.3 (24)	12.5 (1)	23.1 (9)
Tetracycline	47.5 (29)	62.5 (5)	20.5 (8)
Rifampin	98.4 (60)	100.0 (8)	

Empty entries in the table indicate that this data is not available.

**Table 6:** The antibiotic susceptibility of common Gram-Negative bacteria [%/(N)].

Antibiotic	*P. aeruginosa*(n=22)	*K. pneumoniae*(n=5)
Cefoperazone sodium sulbactam	95.2 (20)	60.0 (3)
Piperacillin/tazobactam	95.5 (21)	60.0 (3)
Aminotransol	80.0 (16)	60.0 (3)
Ampicillin	6.3 (1)	40.0 (2)
Amoxicillin Clavulanic Acid	6.3 (1)	20.0 (1)
Piperacillin	90.5 (19)	60.0 (3)
Cefazolin	5.9 (1)	20.0 (1)
Ceftazidime	90.9 (20)	20.0 (1)
Ceftriaxone	5.9 (1)	60.0 (3)
Cefepime	95.5 (21)	20.0 (1)
Ertapenem	10.0 (1)	80.0 (4)
Imipenem	90.9 (20)	80.0 (4)
Meropenem	90.9 (20)	80.0 (4)
Amikacin	95.5(21)	80.0 (4)
Gentamicin	93.8 (15)	60.0 (3)
Ciprofloxacin	90.9 (20)	20.0 (1)
Levofloxacin	90.9 (20)	20.0 (1)
Furantoin	0.0 (0)	20.0 (1)
Cotrimoxazole	0.0 (0)	40.0 (2)
Tetracycline	0.0 (0)	40.0 (2)

Methicillin-Resistant *Staphylococci* accounted for 18.1% of all Gram-Positive bacteria, 47 patients infected with Methicillin-Resistant *Staphylococci* were tested for drug sensitivity. 56.5% (39/69) of *S. epidermidis* were MRSE and 37.5% (3/8) of *S. aureus* were MRSA. Among the *Staphylococcus* spp., 33.8% (47/139) were methicillin-resistant, with 83.0% (39/47) being MRSE and 6.4% (3/47) being MRSA. All Methicillin-Resistant *Staphylococci* isolates demonstrated resistance to penicillin and benzoxicillin, while they were fully susceptible to quinupristin/dalfopristin, tigecycline, linezolid, and vancomycin. Moreover, 63.4% were resistant to levofloxacin, and 48.9% were resistant to ciprofloxacin, **Table 7**.

**Table 7:** Sensitivity to Antibiotics of MRSS [%/(N)].

Antibiotic	Sensitive	Resistant	Intermediary
Penicillin	0.0 (0)	100.0 (47)	0.0 (0)
Benzoxicillin	2.1(1)	97.9 (46)	0.0 (0)
Linezolid	100.0 (47)	0.0 (0)	0.0 (0)
Vancomycin	100.0 (47)	0.0 (0)	0.0 (0)
Tigecycline	100.0 (47)	0.0 (0)	0.0 (0)
Gentamicin	55.3 (26)	10.6 (5)	34.0 (16)
Quinupristin/Dafopristin	100.0 (47)	0.0 (0)	0.0 (0)
Ciprofloxacin	38.3 (18)	46.8 (22)	14.9(7)
Levofloxacin	38.3 (18)	59.6 (28)	2.1 (1)
Moxifloxacin	38.3 (18)	17.0 (8)	44.7 (21)
Erythromycin	14.9 (7)	83.0 (39)	2.1 (1)
Clindamycin	34.0 (16)	63.8 (30)	2.1 (1)
Tetracycline	38.3 (18)	61.7 (29)	0.0 (0)
Rifampin	97.9 (46)	2.1 (1)	0.0 (0)

Comparative analysis, as displayed in **Table 8**, revealed that *S. epidermidis* exhibited higher resistance rates to levofloxacin (*χ2 = 35.144, P<0.001*), moxifloxacin (*χ2 = 8.872, P=0.008*), and ciprofloxacin (*χ2 = 16.254, P=0.001*) compared to other corneal isolates. These differences were statistically significant (*P<0.01*). The sensitivity of MRSA, MSSA, MRSE and MSSE to common antibiotics is shown in **Table 9**.

**Table 8:** Comparative analysis of the resistance of major isolated bacteria to commonly used quinolones[N/(%)].

Antibiotic	Staphylococcus epidermidis	S. aureus	P.aeruginosa	Other bacteria	*χ^2^* value	*P* value
Levofloxacin	31 (50.8)	1 (12.5)	2 (9.1)	21 (14.0)	35.144	<0.001
Moxifloxacin	8 (13.1)	1 (12.5)	/	2 (1.8)	8.872	0.008
Ciprofloxacin	26 (42.6)	2 (25.0)	2 (9.1)	13 (17.3)	16.254	0.001

“/” means the P.aeruginosa lack of susceptibility tests for moxifloxacin.

**Table 9:** Sensitivity to Antibiotics of MRSS, MSSA, MRSE, MSSE [%/(N)].

Antibiotic	MRSA(n=3)	MSSA(n=5)	MRSE(n=35)	MSSE(=26)
Penicillin	0.0 (0)	0.0 (0)	0.0 (0)	0.2 (6)
Benzoxicillin	0.0 (0)	1.0 (5)	0.0 (1)	0.6 (16)
Linezolid	1.0 (3)	1.0 (5)	1.0 (35)	1.0 (26)
Vancomycin	1.0 (3)	1.0 (5)	1.0 (35)	1.0 (26)
Tigecycline	1.0 (3)	1.0 (5)	1.0 (35)	1.0 (26)
Gentamicin	0.7 (2)	0.4 (2)	0.5 (18)	0.8 (22)
Quinupristin/Dafopristin	1.0 (3)	1.0 (5)	1.0 (35)	1.0 (25)
Ciprofloxacin	0.7 (2)	0.4 (2)	0.4 (13)	0.7 (17)
Levofloxacin	0.7 (2)	0.8 (4)	0.4 (13)	0.7 (17)
Moxifloxacin	0.7 (2)	0.8 (4)	0.4 (13)	0.7 (17)
Erythromycin	0.0 (0)	0.2 (1)	0.1 (5)	0.3 (8)
Clindamycin	0.0 (0)	0.2 (1)	0.3 (11)	0.5 (13)
Tetracycline	0.7 (2)	0.6 (3)	0.3 (11)	0.7 (18)
Rifampin	1.0 (3)	1.0 (5)	1.0 (34)	1.0 (26)

## Discussion

The cornea, situated in the foremost region of the eye and in direct contact with the external environment, bears susceptibility to bacterial assaults, injuries, and subsequent corneal infections. Within this study, those involved in agriculture face an elevated risk of keratitis due to occupational-related trauma. Given that adult males form the primary workforce segment, they exhibit a considerably higher likelihood of suffering opportunistic trauma compared to other age groups (Xu et al., 2021). Unfortunately, some patients delayed diagnosis and treatment. As a consequence, a portion of these patients’ vision becomes irreparable by the time they consult an ophthalmologist. Literature reports from rural areas in South America, Asia, and Africa have highlighted such cases (Basak et al., 2005; Kumar et al., 2011; Oladigbolu et al., 2013).

Within this study, a total of 434 patients (65.8%) exhibited solely bacterial infection, while 226 patients (34.2%) presented bacterial co-infection alongside other forms of keratitis. It has been observed that mixed infections can be present in up to 20-30% of cases of keratoconjunctivitis (Pate et al., 2006). These bacterial populations settle on the surface of the cornea, assuming the form of biofilms (Urwin et al., 2020). In certain instances, a mutual symbiotic relationship between specific pathogens and certain fungi, such as Candida albicans, can manifest, facilitating the formation of biofilms. These biofilms create an environment conducive to bacterial co-infections, shielding them from the body’s immune defenses and antibiotic interventions.

In most instances, the transition of bacteria residing on the ocular surface, from harmless commensals to pathogenic invaders, occurs following disruptions in the integrity of the corneal epithelium. This, in turn, leads to altered bacterial ecological niches, an upsurge in local bacterial colonization, and subsequent invasion and infection by harmful microorganisms. Such infection damages the local defensive barriers, including the antimicrobial components of the tear film, resulting in heightened bacterial adhesion and invasion. Ultimately, these processes culminate in stromal necrosis, instigating corneal inflammation, and giving rise to ulcer formation (Singh et al., 2022). While the direct relationship between common colds and physical exertion and the development of BK remains uncertain, these factors may contribute to the disease by temporarily compromising the body’s systemic and local defense mechanisms (Tuft et al., 2022).

In our study, ocular trauma emerged as the most prevalent risk factor (37.1%), followed by a history of previous keratitis (10.6%) and ocular surface disease (5.9%). Similarly, ocular trauma is the most common risk factor for infectious keratitis in other studies by Xu et al. (2021) (Xu et al., 2021) and Oliveira-Ferreira et al. (2019) (Oliveira-Ferreira et al., 2019). The majority of ocular trauma cases and corneal foreign bodies can be attributed to workplace accidents, particularly among manual laborers. Exposure to soil and organic matter without adequate ocular protection significantly increases the risk of bacterial infection, particularly among men aged 30-50 years (Singh et al., 2022). In diabetic patients, ocular surface changes resulting from hyperglycemia heighten the risk of BK. The chronic hyperglycemic state of these patients alters the ocular surface microbiota, promoting the colonization of specific bacteria (Li et al., 2019; Zhu et al., 2019).

In this study, the distribution of strains was dominated by 231 (70.0%) Gram-Positive cocci and 69 (20.9%) Gram-Negative bacilli. The distribution of bacterial isolates in our study was consistent with those reported in studies of West Anatolia and England (Yilmaz et al., 2007; Tan et al., 2017; Ting et al., 2018), where *S. epidermidis* (20.9%) and *S. pneumoniae* (12.1%) are the most common Gram-Positive isolates, with *P. aeruginosa* (7.0%) being the most common Gram-Negative isolates among the ocular pathogens. Analysis of data from 2015 to 2022 revealed that each year witnessed the highest positive rate for *S. epidermidis*, with a noticeable upward trend in the proportion of *P. aeruginosa*, alongside no significant change in the proportion of other species. The most common pathogens for BK remain controversial. *Pseudomonas* spp. proved to be the most common pathogen in Malaysia (Termote et al., 2018), Iran (Al-Dhaheri et al., 2016) and Taiwan (Gupta and Ram, 2016), while coagulase-negative *staphylococci* (CoNS) was reported to be the most common in the UK (Tan et al., 2017; Ting et al., 2018) and Australia (Green et al., 2019). The extensive utilization of broad-spectrum antibiotics likely contributes to significant variation in bacterial spectrum and antibiotic resistance over time and across different geographic regions.

It has been suggested that CoNS are opportunistic pathogens, probably because they are a major component of the normal flora of the skin and conjunctival capsule. On one hand, the proximity of these bacteria to the skin may heighten the risk of corneal tissue infection. On the other hand, contamination during corneal scraping sampling can lead to an increased detection rate of CoNS (Termote et al., 2018). The bacterial isolates commonly observed in this study included *Staphylococcus* spp., *Pseudomonas* spp., and *Streptococcus* spp. These findings align with research conducted in the United States (Lin et al., 2019), the United Kingdom, and Canada.

The culture-positivity rate observed in this study, reaching 61.04%, fell within the range of previously reported rates, which have ranged from 32.60% to 79.20% (Amatya et al., 2012; Tan et al., 2017). The specific type of pathogenic microorganism can vary depending on the patient’s susceptibility to risk factors and the geographic region. Despite these variations in the causative microorganisms of microbial keratitis (BK) across different locales, one consistent finding is the higher proportion of infections caused by Gram-Positive bacteria (ranging from 48% to 89%) compared to Gram-Negative bacteria (ranging from 11% to 50%) (Ung et al., 2019). Regarding the detection rates of Gram-Positive cocci and Gram-Negative bacilli over time, no significant trend was observed for Gram-Positive cocci, while there was an increasing trend in the detection rate of Gram-Negative bacilli (*Rs=0.743, P=0.035*). Notably, studies conducted in the UK (Ting et al., 2018) and Iran (Al-Dhaheri et al., 2016) revealed different trends. Specifically, an upward trend in Gram-Positive cocci detection and a downward trend in Gram-Negative bacilli detection were observed. Due to the geographical variances in pathogenic bacterial profiles, continuously monitoring the changes in regional pathogenic bacterial profiles serves as a valuable guide for clinical treatment.

*In vitro* drug susceptibility tests revealed that Gram-Positive bacteria exhibited high sensitivity to linezolid, vancomycin, tigecycline, quinupristin/dalfopristin, and rifampin, with susceptibility rates exceeding 98%. Ciprofloxacin, levofloxacin, and moxifloxacin showed sensitivity rates of 65.0%, 74.1%, and 71.2% respectively. On the other hand, Gram-Negative bacteria demonstrated greater sensitivity to cefoperazone sodium/sulbactam, piperacillin/tazobactam, meropenem, amikacin, ceftazidime, and cefepime, with rates above 85%. However, their sensitivity to levofloxacin and ciprofloxacin was 77.6% and 68.8% respectively, with only 16.7% sensitivity to cefazolin and ampicillin. In Southern India, there was a noteworthy increase in fluoroquinolone resistance among *S. aureus* and *P. aeruginosa* isolated from BK patients from 2002 to 2013, with *S. aureus* resistance to ofloxacin rising from 11.1% to 66.7% (Lalitha et al., 2017). In the present study, *S. epidermidis* exhibited significantly higher resistance rates to commonly used quinolones compared to other isolates, and these differences were statistically significant (*P<0.01*). Two cases of *P. aeruginosa* resistant to fluoroquinolones were identified, one of which displayed multidrug resistance. The susceptibility of Gram-Negative bacteria to fluoroquinolones in the present study was 74.1%, with Gram-Negative bacteria overall demonstrating a susceptibility rate of 77.6%. This is consistent with the decreasing trend observed worldwide. Notably, the susceptibility of *P. aeruginosa* to fluoroquinolones was 90.9%, aligning with the global average susceptibility (Dinesh, 2018). It is important to acknowledge that resistance to fluoroquinolones does exist, with occasional cases of *P. aeruginosa* resistance. Fluoroquinolones are widely employed as monotherapy for empiric treatment of BK due to their broad spectrum of activity (Milder et al., 2012). However, the continuous usage has contributed to the emergence of resistance in this class of drugs. A recent study pointed out that the greatest resistance to antibiotics was observed with fluoroquinolones (Sahoo et al., 2023). Ophthalmologists should remain vigilant regarding the evolving trends in pathogenic bacterial distribution and resistance patterns, adapting the diagnosis to local conditions and selecting appropriate antibiotics accordingly.

All isolates of Methicillin-Resistant *Staphylococci* demonstrated resistance to penicillin and benzoxicillin, while displaying 100% susceptibility to vancomycin, linezolid, tigecycline, and quinupristin/dalfopristin. According to the Centers for Disease Control (CDC), approximately 2 million individuals are infected with drug-resistant microorganisms annually (Austin et al., 2017). When addressing Methicillin- and benzoxicillin-resistant *S. aureus*, vancomycin has become the primary therapeutic agent (Saillard et al., 2018). The Antibiotic Resistance Monitoring in Ocular Microorganisms study in April 2020 revealed high rates of methicillin resistance and MDR among *S. aureus* and CoNS isolates, though oxacillin/methicillin resistance was slightly reduced among *S. aureus* isolates and unchanged among CoNS isolates (Asbell et al., 2020). Our findings align with previous reports stating that MRSA or MR-CoNS strains may exhibit resistance to multiple drugs. In our study, one in three *S. aureus* isolates and one in two CoNS isolates demonstrated resistance to methicillin. The data from this study, as well as previous studies, suggest that oral linezolid could be a viable treatment option in cases of methicillin-resistant *staphylococcal* keratitis progressing to endophthalmitis, due to its potent antibacterial activity against this pathogen.

The present study has certain limitations that should be acknowledged. Firstly, it relied on a retrospective analysis of available medical records, which may have introduced inherent biases and limitations in terms of data collection and accuracy. Secondly, there was a potential bias in the selection of cases, which could have influenced the representation and generalizability of the findings. Given that keratitis is a challenging condition to treat, there was limited improvement observed in the patient’s visual function during their hospital stay. Unfortunately, due to the nature of this study, the final visual acuity of the patient after successful treatment could not be determined. Lastly, it is important to note that some patients with mild symptoms were excluded from the study after receiving empirical topical antibiotic treatment in the outpatient department. These exclusions might have impacted the overall population under investigation and could potentially affect the interpretation of the results. It is crucial for future research to address these limitations and consider prospective study designs to provide more robust and comprehensive insights into the topic at hand.

## Conclusions

When dealing with patients who have a history of vegetative trauma, it is crucial to consider the possibility of bacterial keratitis (BK) in addition to focusing on fungal keratitis. The distribution of bacterial strains is primarily comprised of Gram-Positive cocci and Gram-Negative bacilli. Among the Gram-Positive bacteria, the most frequently encountered species are *S. epidermidis*, whereas *P. aeruginosa* is the most common Gram-Negative species. When it comes to combating Gram-Positive bacteria, vancomycin, linezolid, and rifampicin prove to be effective antimicrobial agents. In the treatment of Gram-Negative infections, third-generation cephalosporins demonstrate superior sensitivity compared to their first and second-generation counterparts. As an initial empirical treatment for severe cases of bacterial keratitis (BK) and cases that fail to respond to fourth-generation fluoroquinolones in the community, a reasonable approach would be to combine vancomycin and tobramycin. A better management of bacterial keratitis can be achieved through a comprehensive understanding of the local etiology and patterns of antibacterial drug susceptibility.

## Data availability statement

The raw data supporting the conclusions of this article will be made available by the authors, without undue reservation.

## Ethics statement

The studies involving humans were approved by The Ethics Committee of the Affiliated Hospital of Yunnan University approved the application for waiver of informed consent (No. 2023176). The studies were conducted in accordance with the local legislation and institutional requirements. Written informed consent for participation was not required from the participants or the participants’ legal guardians/next of kin in accordance with the national legislation and institutional requirements.

## Author contributions

RG: Investigation, Methodology, Writing – original draft. JY: Conceptualization, Writing – review & editing. YY: Conceptualization, Investigation, Methodology, Writing – original draft. YC: Investigation, Methodology, Writing – original draft. YX: Investigation, Writing – original draft. PX: Conceptualization, Writing – review & editing. MD: Investigation, Methodology, Writing – original draft. MH: Data curation, Funding acquisition, Writing – original draft. YW: Data curation, Formal Analysis, Writing – original draft. YX: Formal Analysis, Writing – original draft. HK: Data curation, Formal Analysis, Funding acquisition, Supervision, Writing – review & editing. HL: Conceptualization, Funding acquisition, Resources, Supervision, Writing – review & editing.
